# Predictors of yoga use among internal medicine patients

**DOI:** 10.1186/1472-6882-13-172

**Published:** 2013-07-13

**Authors:** Holger Cramer, Romy Lauche, Jost Langhorst, Anna Paul, Andreas Michalsen, Gustav Dobos

**Affiliations:** 1 Department of Internal and Integrative Medicine, Kliniken Essen-Mitte, Faculty of Medicine, University of Duisburg-Essen, Essen, Germany; 2 Immanuel Hospital Berlin, Department of Internal and Complementary Medicine, Berlin, Germany

**Keywords:** Yoga, Complementary therapies, Internal medicine

## Abstract

**Background:**

Yoga seems to be an effective means to cope with a variety of internal medicine conditions. While characteristics of yoga users have been investigated in the general population, little is known about predictors of yoga use and barriers to yoga use in internal medicine patients. The aim of this cross-sectional analysis was to identify sociodemographic, clinical, and psychological predictors of yoga use among internal medicine patients.

**Methods:**

A cross-sectional analysis was conducted among all patients being referred to a Department of Internal and Integrative Medicine during a 3-year period. It was assessed whether patients had ever used yoga for their primary medical complaint, the perceived benefit, and the perceived harm of yoga practice. Potential predictors of yoga use including sociodemographic characteristics, health behavior, internal medicine diagnosis, general health status, mental health, satisfaction with health, and health locus of control were assessed; and associations with yoga use were tested using multiple logistic regression analysis. Odds ratios (OR) with 95% confidence intervals (CI) were calculated for significant predictors.

**Results:**

Of 2486 participants, 303 (12.19%) reported having used yoga for their primary medical complaint. Of those, 184 (60.73%) reported benefits and 12 (3.96%) reported harms due to yoga practice. Compared to yoga non-users, yoga users were more likely to be 50–64 years old (OR = 1.45; 95%CI = 1.05-2.01; P = 0.025); female (OR = 2.45; 95%CI = 1.45-4.02; P < 0.001); and college graduates (OR = 1.61; 95%CI = 1.14-2.27; P = 0.007); and less likely to currently smoke (OR = 0.61; 95%CI = 0.39-0.96; P = 0.031). Manifest anxiety (OR = 1.47; 95%CI = 1.06-2.04; P = 0.020); and high internal health locus of control (OR = 1.92; 95%CI = 1.38-2.67; P < 0.001) were positively associated with yoga use, while high external-fatalistic health locus of control (OR = 0.66; 95%CI = 0.47-0.92; P = 0.014) was negatively associated with yoga use.

**Conclusion:**

Yoga was used for their primary medical complaint by 12.19% of an internal integrative medicine patient population and was commonly perceived as beneficial. Yoga use was not associated with the patients’ specific diagnosis but with sociodemographic factors, mental health, and health locus of control. To improve adherence to yoga practice, it should be considered that male, younger, and anxious patients and those with low internal health locus of control might be less intrinsically motivated to start yoga.

## Background

Yoga has its roots in Indian philosophy and has been a part of traditional Indian spiritual practice for around 5000 years [1]. Yoga comprises advice for ethical lifestyle as well as spiritual practice [2]. While the ultimate goal of yoga has been described as uniting mind, body and spirit [2], yoga has also been used to promote physical and mental well-being. In North America and Europe, yoga is most often associated with physical postures (asanas), breathing techniques (pranayama), and meditation (dyana) [1]. It differs from purely gymnastic exercises in that the practitioner focuses his mind on the postures with inner awareness and a meditative focus of mind [3,4]. In recent years, yoga is gaining increased popularity as a therapeutic practice: in 2002, more than 10 million Americans practiced yoga as a means of improving their health, and by 2007, more than 13 million [5,6].

Yoga has been shown to improve a variety of physical health conditions such as low back pain [7], fibromyalgia [8], arthritis [9], or irritable bowel syndrome [10]. Yoga can reduce risk factors for cardiovascular disease [11] and improve risk profiles in adults with type 2 diabetes mellitus [12]. Other conditions such as asthma seem to be relatively little improved by yoga interventions [13].

Despite the therapeutic value of yoga for internal medicine patients, little is known about factors that are associated with yoga use and barriers to practice in this patient population. Based on a representative survey, Birdee et al. [14] characterized yoga users as being more likely to be female, young, and well-educated. Yoga users more often suffered from musculoskeletal disorders than non-users; and yoga was most commonly used to treat these disorders. Higher yoga use further was associated with asthma, while hypertension and chronic obstructive lung disease were associated with lower use. An earlier survey also showed an association of yoga use with lung disease and back pain [15]. However, while these surveys were representative for the adult population, characteristics of yoga users have hardly been investigated in patient populations.

The aim of this analysis was to identify sociodemographic, clinical, and psychological predictors of yoga use for the patient’s primary medical complaint among internal medicine patients. The analysis was based on sociodemographic variables, medical diagnosis, mental health, satisfaction, and health locus of control [16] that describes health behavior as a function of control beliefs [16]: an individual’s belief about control of health outcomes can be classified as either internal (health depends on the individual’s attributes or behavior) or external (health depends on the behavior of other people, fate or luck) [16,17]. It has been shown that an internal health locus of control is associated with carrying out recommended health behaviors such as exercise behavior [18,19] or weight management [20].

To identify predictors for therapeutic yoga use, a cross-sectional analysis was conducted in a mixed internal medicine population. Based on the findings in the US general population, it was hypothesized that yoga use for the patient’s primary medical complaint would be higher in female, young, and well-educated patients. Associations of yoga use were further expected for patients with specific medical conditions such as musculoskeletal conditions and lung diseases. Based on the findings on other categories of health behavior, it was further hypothesized that higher yoga use would be found in patients with higher internal health locus of control.

## Methods

### Design

The survey was part of the internal quality assurance of the Department for Internal and Integrative Medicine, Essen, Germany. All data were assessed as paper-based self-rated questionnaires except for main diagnosis, age, and gender that were taken from the patient’s medical record. Questionnaires were handed out at admission and collected in by the receptionist. The information on medical diagnosis and prior complementary therapies use were also used for developing the individual treatment plan.

### Ethics approval

The survey was conducted in compliance with the Helsinki Declaration. No approval was required from an ethics committee according to the German law^a^.

### Participants

All patients being referred to a Department for Internal and Integrative Medicine between January 2001 and January 2004 were asked to complete the survey immediately upon admission. All patients were diagnosed with an internal medicine condition [21]. All included patients gave written informed consent. All data were de-identified and analyzed anonymously.

### Yoga use

Using the Freiburg Questionnaire on Attitudes on Naturopathy it was assessed which complementary therapies patients had used before to treat their primary medical complaint [22]. For this analysis, only the question on yoga use was evaluated: Have you ever used yoga for your primary medical complaint? (response choice: yes/no). Those patients that reported to have used yoga for their primary medical complaint were queried about helpfulness: How helpful was yoga for your primary medical complaint? (response choices: helpful/not helpful/harmful). Answers were categorized as helpful (yes, no) and harmful (yes, no).

### Potential predictors of yoga use

#### Sociodemographic characteristics

Linear sociodemographic variables were categorized in order to be able to compute odds ratios between yoga-users and non-users. Age (18–29, 30–39, 40–49, 50–64, ≥65 years), sex, education (less than college, college graduate), employment (full-time, part-time, unemployed), and family status (in relationship, not in relationship), were used as possible predictors together with health behaviors including smoking status (current smokers, past smoker, non-smokers), alcohol intake (abstainers, less than twice weekly, at least twice weekly), fast food intake (abstainers, less than twice weekly, at least twice weekly).

#### Clinical characteristics

The patients’ main diagnosis was assessed by the transferring doctor according to ICD-10 [23]. For this analysis, diagnoses were categorized as a) osteoarthritis, b) arthritis, c) fibromyalgia, d) spinal pain, e) headache, f) other pain, g) hypertension, h) ischemic cardiac disease, i) irritable bowel syndrome, j) inflammatory bowel disease, k) lung diseases, l) other, more rare conditions.

General health status was assessed on a 5 point scale and categorized as good, very good or excellent versus poor or fair.

#### Mental health

Anxiety and depression were assessed using the Hospital Anxiety and Depression Scale (HADS) [24]. Scores ranging from 8 to 10 were defined as subthreshold anxiety or depression while scores > 10 were defined as threshold anxiety or depression [25]. In this sample, Cronbach’s α was 0.81 for the anxiety subscale and 0.84 for the depression subscale.

#### Satisfaction with health and life in general (FLZ)

Satisfaction with health and life in general were assessed using one 5-point Likert item each from the questionnaire for life satisfaction (FLZ) [26]. The endpoints were 1 = very unsatisfied and 5 = very satisfied. The higher the score the more satisfied the patient was with health and life in general. Every patient was categorized as having either high (i.e. above median) or low (i.e. below median) satisfaction with health and satisfaction with life in general.

#### Health locus of control

Health locus of control was assessed using the GKÜ [27], a 9-item German modified short-form of the multidimensional health locus of control scale, the most commonly used scale to assess health locus of control [17]. Response choices ranged from “strongly disagree” to “strongly agree”. The instrument assesses 3 dimensions of health locus of control beliefs (3 items each): internal (high perceived own influence on health status), external-social (health status perceived as controlled by others) and external-fatalistic (health status perceived as depending on luck or destiny). Cronbach’s α was 0.74 for internal, 0.46 for external-social, and 0.53 for external-fatalistic health locus of control. For every patient, each dimension was categorized as either high (i.e. above median) or low (i.e. below median).

#### Statistical analysis

Chi square tests were used to compare sociodemographic, clinical, and psychological characteristics between patients who ever used yoga for their primary medical complaint and those who did not. A p-value of ≤0.05 was considered statistically significant. Independent predictors of therapeutic yoga use were identified using multiple logistic regression analysis. A backward stepwise procedure with a Wald statistic p-value of ≤0.05 was used. In order to be able to compare the individual influence the different variables had on yoga use, continuous variables were categorized and adjusted odds raios with 95% confidence intervals were calculated. Among the potential factors that were entered in the initial regression model, only factors were selected that were associated with yoga use at a p-value of ≤0.10 in univariate analysis. Statistical analysis was performed using IBM SPSS® software (release 20.0, IBM, USA).

## Results

Of the 2804 patients being referred during the 3-year study period, 2486 agreed to participate in the survey (response rate 88.66%). Of the respondents, 325 patients (13.07%) did not answer the question on yoga use. Therefore, the analysis was based on a sample of 2161 patients. In this sample, the amount of missing values ranged from 0.0% for diagnosis, age, and gender to 4.8% for one item of the GKÜ. Overall, 1.8% of the values were missing. Out of 2161 patients, 1715 (79.4%) were female, mean age was 52.9 ± 14.1. Most patients were diagnosed with chronic pain; the most commonly diagnosed conditions were spinal pain (n = 412; 19.1%), headache (n = 257; 11.9%), and fibromyalgia (n = 249; 11.5%). Five hundred eighty-one patients (29.9%) were high school graduates; 569 (26.3%) were full-time employed; and 341 (15.8%) were part-time employed. One thousand two hundred sixty-two patients (58.4%) were in a relationship. Regarding health behavior, 1834 (84.9%) regularly consumed fast food; 357 (16.5%) regularly consumed alcoholic beverages; and 299 (13.8%) currently smoked.

Three hundred and three patients (12.19%) reported having used yoga for their primary medical complaint. Of those, 184 (60.73%) reported that yoga had been helpful and 12 (3.96%) reported that yoga had been harmful.

In univariate analysis, yoga use was higher with older age while lower at age beyond 64. Yoga users were more often female than non-users, and more often college graduates than non-users. Yoga users were less often currently smoking. Headache and inflammatory bowel disease were associated with more yoga use. Patients with threshold anxiety and/or subthreshold depression more often used yoga. Patients with high internal health locus of control more often used yoga as well as did patients with low external-social and/or external-fatalistic health locus of control (Table 1).

**Table 1:** **Sociodemographic**, **clinical**, **and psychological characteristics of patients who used yoga for their primary medical complaint** (**yoga users**) **and those who did not** (**yoga non**-**users**)

	**Yoga non-users (n = 1858)**	**Yoga users (n = 303)**	**P-value**
	**N (%)**	**N (%)**	
Age group			
Less than 30	111 (5.97%)	14 (4.62%)	0.426
30 to 39	247 (13.29%)	47 (15.51%)	0.320
40 to 49	361 (19.43%)	65 (21.45%)	0.436
50 to 64	708 (38.11%)	138 (45.55%)	0.016
65 or greater	431 (23.20%)	39 (12.87%)	<0.001
Gender			
Female	1449 (77.99%)	266 (87.79%)	<0.001
Education			
High school graduate	474 (26.06%)	107 (36.03%)	0.001
Employment			
Full-time	477 (26.75%)	92 (31.19%)	0.121
Part-time	289 (16.21%)	52 (17.63%)	0.553
In relationship	1100 (59.88%)	162 (54.18%)	0.066
Health behavior			
Current smoker	272 (21.40%)	27 (14.21%)	0.021
Past Smoker	404 (31.79%)	69 (36.32%)	0.214
Alcohol abstainer	764 (41.73%)	109 (36.09%)	0.067
Alcohol at least twice weekly	308 (16.82%)	49 (16.23%)	0.868
Fast food abstainer	78 (4.30%)	14 (4.68%)	0.760
Fast food at least twice weekly	1573 (84.66%)	261 (86.13%)	0.785
Main diagnosis			
Osteoarthritis	176 (9.47%)	22 (7.26%)	0.238
Arthritis	126 (6.78%)	15 (4.95%)	0.260
Fibromyalgia	207 (11.14%)	42 (13.86%)	0.174
Spinal pain	364 (19.59%)	48 (15.84%)	0.134
Headache	208 (11.19%)	49 (16.17%)	0.016
Other pain	206 (11.09%)	26 (8.58%)	0.229
Hypertension	62 (3.98%)	9 (2.97%)	0.863
Ischemic cardiac disease	22 (1.18%)	2 (0.66%)	0.564
Irritable bowel syndrome	56 (3.01%)	12 (3.96%)	0.288
Inflammatory bowel disease	85 (4.57%)	23 (7.59%)	0.032
Lung diseases	92 (5.91%)	18 (5.94%)	0.480
Others	254 (13.67%)	36 (11.88%)	0.467
Health status			
Good, very good or excellent	310 (16.80%)	48 (15.84%)	0.802
Mental health			
Subthreshold anxiety	471 (25.75%)	80 (27.03%)	0.668
Threshold anxiety	620 (33.90%)	130 (43.92%)	0.001
Subthreshold depression	423 (23.13%)	93 (31.21%)	0.003
Threshold depression	410 (22.42%)	57 (19.13%)	0.227
Satisfaction			
High satisfaction with health	545 (29.64%)	88 (29.14%)	0.892
High satisfaction in general	928 (50.52%)	138 (46.00%)	0.152
Health locus of control			
High internal	707 (38.91%)	165 (55.18%)	<0.001
High external-social	926 (51.05%)	110 (37.29%)	<0.001
High external-fatalistic	861 (47.60%)	113 (38.18%)	0.003

In multiple logistic regression analysis, yoga use for the patient’s primary medical complaint was independently associated with female gender, an age of 50–64 years and higher education (Table 2). Regarding health behavior, the only association found was that yoga users were less likely to currently smoke. Yoga users were more likely to suffer from manifest anxiety while no association with internal medicine diagnosis was found. Yoga use was independently associated with high internal health locus of control and with low external-fatalistic health locus of control (Table 2).

**Table 2:** **Independent predictors of yoga use for the patient**’**s primary medical complaint**

**Predictor variable**	**P**	**Adjusted odds ratio**	**95% confidence interval**
Age 50-64	0.025	1.45	1.05-2.01
Female gender	<0.001	2.45	1.45-4.02
Education: College graduate	0.007	1.61	1.14-2.27
Current smoker	0.031	0.61	0.39-0.96
Threshold anxiety	0.020	1.47	1.06-2.04
Internal health locus of control	<0.001	1.92	1.38-2.67
External-fatalistic health locus of control	0.014	0.66	0.47-0.92

## Discussion

In this survey, 12.19% of internal medicine patients reported having used yoga for their primary medical complaint. The majority of patients found yoga to be helpful in dealing with their complaints and only a small minority reported harms associated with yoga practice. Yoga users were more likely to be female, in their fifties to mid-sixties and well educated. They smoked less than non-users. Yoga use was associated with above-threshold levels of anxiety but not with internal medicine diagnosis. Patients who used yoga for their primary medical complaint tended to have high internal health locus of control and low external-fatalistic health locus of control.

While no data on the rate of yoga use in the European population is available, the prevalence of yoga use found in this survey is higher than that found in the general population where prevalence ranged from 5.1% [5,14] to 7.5% [15]. A recent survey in breast cancer patients however reported even higher prevalence of yoga use after cancer diagnosis [28]. While differences between settings limit the comparability of these results, this might indicate that specific patient groups tend to try yoga more often than the general population. The finding that yoga use is higher among well-educated non-smoking persons is in line with prior surveys in the general education [14,15] and in breast cancer patients [28]. Female gender also has commonly been associated with higher yoga use [14,15]. While no association with age was found in breast cancer patients, yoga users in the general population tended to be younger than non-users, a finding that contradicts the results of the present survey: in the 2002 National Health Interview Survey, age of 40 years or higher was significantly associated with lower yoga use compared to the age group of 30 years or lower [14]. Comparably, an earlier survey found significantly higher rates of yoga use in the age group of 34–53 years when compared to those aged 54 years or higher [15].

Yoga use has been associated with a variety of health conditions in the general population, mainly musculoskeletal or mental disorders [14], and lung disease [15]. Interestingly, in the current survey, that focused specifically on internal medicine patients, yoga use was associated with anxiety but not with internal medicine diagnosis. Yoga has been shown to have beneficial effects on a variety of internal medicine conditions including spinal pain [7,29], fibromyalgia [8], and arthritis [9]. While the prior surveys suggest that these are the most common conditions for which yoga is used [14,15], this is not reflected in the current patient-based survey. As other conditions such as asthma [13] are less likely to benefit from yoga use, internal medicine patients’ decision to try yoga to cope with their conditions does not seem to be evidence-based. Providing more detailed information about evidence of effectiveness of yoga for specific health conditions could improve patients’ possibility for informed decision making.

Health locus of control was a strong predictor of yoga use for the patients’ primary medical complaints. Yoga use was higher in patients who had high internal health locus of control, that is the belief that health depends on the persons own behavior; and low external-fatalistic health locus of control, that is the belief that health depends on fate on luck [16,17]. As yoga is an active coping strategy that depends on the patients’ motivations and actions, this association is reasonable. It is in line with prior studies that found associations of exercise behavior with higher internal and lower external health locus of control [18,19]. General use of complementary therapies has also been found to be associated with high internal health locus of control [30]. It should be noted that internal and external health locus of control are generally uncorrelated with each other or only slightly negatively intercorrelated [17]. Therefore one patient could theoretically have both high internal and high external health locus of control.

The results of this study are limited by the single-center setting that might limit generalizability of the results. The results might therefore only apply to German internal medicine patients that have a general interest in integrative medicine. The Department of Internal and Integrative Medicine Essen, Germany, is a model institution that treats patients with internal diseases using a combination of conventional and evidence-based complementary therapies [21]. While the department might specifically attract patients who are generally interested in internal medicine, patients have to be referred to the department by their general practitioners and costs are covered by the German statutory and many private health insurances. A further limitation is that the data has been collected several years ago. As the rate of yoga use has been shown to steadily increase in the USA [5,6,], a comparable development might be considerable for Germany. While no data on yoga use in Germany are available, it seems likely that this analysis underestimates the total rate of actual yoga use. The survey did not assess reasons for yoga use, attitudes towards yoga, specific yoga style, or frequency of yoga use. Subjective efficacy and safety of yoga use were assessed but the perceived benefits or harms were not investigated in depths.Moreover, due to the design of the questionnaire, patients were unable to rate yoga as both helpful and harmful. Internal consistency of the instruments was good for the HADS, acceptable for the internal scale of the GKÜ, but poor for the external-fatalistic scale. Therefore, external-fatalistic health locus of control as a predictor of yoga use has to be interpreted with care. As single items from the FLZ were used instead of the complete instrument, psychometric properties are unclear for satisfaction. Race, religion, body mass index, and other factors that have been shown to be associated with yoga use in earlier studies were not assessed in the current survey. Despite these limitations, the current study can enrich earlier findings on predictors of yoga use in other populations [14,15,28].

Future research should investigate predictors of yoga use for the patient’s primary medical complaint in a more representative patient sample and focus on reasons for use and barriers to use. Investigation of knowledge about evidence of effectiveness for yoga and on yoga in general in this patient population could improve informed decision making about use or non-use of yoga as a coping strategy.

## Conclusion

Yoga was practiced for the patients’ primary medical complaints by 12.09% of an internal medicine patient population and was commonly perceived as beneficial. Yoga use was not associated with the patients’ specific diagnosis but with sociodemographic factors, mental health, and health locus of control. To improve adherence to yoga practice in research and clinical practice, it should be considered that males, younger patients, smokers, anxious patients, and those with low internal health locus of control might be less intrinsically motivated to start yoga and might need additional information.

## Endnotes

^a^In Germany, the need for ethical approval is only statutorily regulated for clinical trials on drugs and medical devices, and for stem cell research.

http://www.gesetze-im-internet.de/amg_1976/__40.html [German only].

http://www.gesetze-im-internet.de/bundesrecht/mpv_2002/gesamt.pdf [German only].

http://www.gesetze-im-internet.de/bundesrecht/stzg/gesamt.pdf [German only].

The Medical Association's Professional Code of Conduct (Berufsordnung für die in Deutschland tätigen Ärztinnen und Ärzte der Bundesärztekammer) regulates the need for further ethical approval of other research conducted by physicians and states that research projects that interfere with a human’s physical or psychological integrity or uses bodily materials or data that are relatable to a specific human need to be approved by an ethics committee. This was not the case in this survey as all data were de-identified and analyzed anonymously.

http://www.bundesaerztekammer.de/page.asp?his=1.100.1143 [German only]

## Competing interests

The authors declare that they have no competing interests.

## Authors’ contributions

HC was responsible for conception and design, analysis and interpretation of the data, and drafted the manuscript. RL participated in conception and design, analysis and interpretation of the data, and critically revised the manuscript. JL, AP, AM, and GD participated in conception and design, acquisition of data, and critically revised the manuscript. All authors read and approved the final manuscript.

## Pre-publication history

The pre-publication history for this paper can be accessed here:

http://www.biomedcentral.com/1472-6882/13/172/prepub
